# Changes in Catastrophic Health Expenditures Depending on Health Policies in Turkey

**DOI:** 10.3389/fpubh.2020.614449

**Published:** 2021-01-07

**Authors:** Guvenc Kockaya, Gülpembe Oguzhan, Zafer Çalşkan

**Affiliations:** ^1^ECONiX, Samsun, Turkey; ^2^Department of Health Management Ondokuz Mayis University, Samsun, Turkey; ^3^Department of Economics, Hacettepe University, Ankara, Turkey

**Keywords:** catastrophic expenditure, health expenditure, out-of-pocket expenditure, Turkey, health transformation plan

## Abstract

Without any financial protection out of pocket health expenses are essential both because their increase causes difficulties in accessing higher quality health services for households and more importantly because it complicates access to most basic health services. As a result of the Health Transformation Program in practice in the Turkish healthcare system since 2003, significant changes have been done in all layers of the health system. Turkish Statistics Institute (TurkStat) publishes the ratio of households that bear catastrophic health expenditures since 2002. According to TurkStat data, the ratio of households with catastrophic expenditure has fallen from 0.81% in 2002 to 0.17% in 2011 with the health transformation project. However, it has started to rise since 2012 and has reached 0.31% in 2014. This study aims to evaluate the expenditure items that may have caused the rise of the ratio of households with catastrophic health expenditures since 2012, which had previously dropped with the Health Transformation Program that has caused fundamental changes in health policies. Methodology and definitions presented in the article named “Distribution of health payments and catastrophic expenditures: Methodology” by Ke Xu published by the World Health Organization in 2005 have been used. Percentages of health expenditure items among the total expenditure of households with positive health expenditure and households with catastrophic health expenditure between 2007 and 2014 have been evaluated using descriptive analysis. Findings have been interpreted in light of the health policies in practice between 2007 and 2014. An overview of the impact of the health policies reveals that medicine expenditures have decreased both for household and public health expenditures. Despite the impact of policies on the pharmaceutical industry was criticized by the industry, the positive impact can be seen by the decrease in the spending on medicine for households spending on health. Hospital service with positive health expenditure is seen to decrease health expenditure. The reasons for the increase in households with catastrophic health expenditure need further research. As a result, the study strives to discuss the possible policy reasons for the observed effects.

## Introduction

While access to healthcare is a basic human right, according to the World Health Organization's Constitution, people with disabilities face several barriers in their effort to access healthcare services and report higher unmet healthcare needs, compared to people with no disabilities (1). One of the important barriers is financial inequity that affects health disparities. All health care systems should provide financial protection for patients. This means that households do not face catastrophic levels of health spending as a result of using healthcare. Therefore “Financial Protection in health” is becoming an increasingly important policy goal for every health system (2–5). Also ensuring financial protection (FP) against health expenditures is a key component of Sustainable Development Goal (SDG) 3.8, which aims to achieve Universal Health Coverage (UHC) (6). In the 2000 report of World Health Organization (WHO) equal finance definition in health systems have been used as a reference and started as; “Risk borne by each household stemming from healthcare expenditures shall be distributed concerning the capability to pay instead of risking disease. Which is to say that health systems shall guarantee financial protection for everyone to ensure equal financing.” WHO report especially states that a health system is unfair if it is causing individuals to impoverish by purchasing health services they need or it prevents them from getting the health services they need due to high costs (7).

The 2011 World Health Statistics showed that health spending in many low-and middle-income countries financed the bulk of households' service payments through out-of-pocket payments (2, 8). Health spending in many developing countries, including the Middle East, most Asian countries, and the North Africa region, is funded mostly through out-of-pocket spending by households (9). Out of pocket health expenses are important both because their increase causes difficulties in accessing higher quality health services for households and more importantly because it complicates access to most basic health services. One of the most important problems that arise is the harm to the “social benefit” principle (10).

Households that are not provided with adequate financial protection face the risk of being exposed to unforeseen large medical expenses if they become ill (3, 11). If these expenses are covered out of pocket, the economy of the household is negatively affected by this event (12). Every year, 100 million people face poverty because of high out-of-pocket spending on health care, and 150 million people are in catastrophic health spending (4). Therefore, protection of households against catastrophic health expenditure has emerged as a leading policy goal for global health. Recently, the WHO, the World Bank, and the United Nations renewed their calls that there should be equality in health and that no one should be impoverished by catastrophic health spending (13).

Financial barriers and their related catastrophic health expenditures are lead to unmet health needs, postpone receiving appropriate and timely health care, delay in medicines use, and hospitalizations. In the determination of catastrophic health expenditures, there are two approaches: the expenditure approach and the income approach. According to the expenditure approach, catastrophic health expenditure occurs when the out of pocket health expenses exceed a certain ratio of the total expenditure (when a threshold value is exceeded) apart from basic expenditures done to sustain the lives of individuals. According to the income approach (capacity to pay), catastrophic health expenditure occurs when out-of-pocket health expenses exceed a certain percentage of the household income (14, 15). Discussions on whether to use total household income or total disposable income as the denominator in calculations continue. Within this framework, the World Health Organization names total disposable income as the capacity to pay and defines it as the value left after expenses for basic needs (mostly food expenses) are deducted from the total income. In studies within this scope, the denominator is generally the value left after food expenses are deducted from the total income (16, 17).

In the income approach, the level of a certain percentage is open to debate. Studies are assuming this level from 5 to 20% of total income in the literature (18–21). While the said limit is still debated in the literature, generally the limit defined as a “fair financing” amount by the World Health Organization is accepted. In the calculation of catastrophic impact and impoverishing impact households exposed to following the methodology defined by Xu and WHO (22), it is especially punctuated that the ratio of out-of-pocket health expenditures to the ability to pay (capacity) is used.

Although the level of expenditure that defines catastrophe is still widely debated, there is an understanding that even low levels of expenditure on health care can tip a household to poverty depending on the household's income (14). To calculate the ability to pay, according to the study by Çinaroglu and Sahin (23), initially, equivalent household size (size) shall be calculated. Equivalent household size enables comparisons between households of different sizes and combinations, with consideration to differences of adults-children combinations of households. For this purpose, the equivalency scale is calculated to determine how many adults (number of equivalent individuals) the household size corresponds to and then the poverty level shall be calculated. It is stated that poverty level is defined as the average food expenses of equivalent individuals of households that spend 45–55% of total income for food; sustenance level is determined based on equivalent household size and households with household expenses under the poverty level defined according to equivalent household size are considered “poor.” To find those with catastrophic health expenses, households with health expenses to the ability to pay ratio of 40% or higher are considered as “households with catastrophic health expenses.”

TurkStat publishes the ratio of households that bear catastrophic health expenditures since 2002. In the survey information on the socioeconomic status of the household in the first visit before the survey, a month is received and an expenditure book is left. During the visits within the survey month, data on consumption expenses of the household such as food, clothing, health, transport, communication, education, culture, recreation, housing, household goods, etc in the survey month are compiled. In the final visit at the end of the survey month, data on household members' employment and their income within the survey month is compiled. In short, according to TurkStat data, the ratio of households with catastrophic expenditure has fallen from 0.81 in 2002 to 0.17% in 2011 with the health transformation project. However, it has started to rise since 2012 and has reached 0.31% in 2014 (24).

This study aims to investigate the expenditure items that may have caused the rise of the ratio of households with catastrophic health expenditure since 2012, which had previously dropped with the Health Transformation Program that has caused fundamental changes in health policies.

## Materials and Methods

### Data

We used the nationally representative Household Budget Survey implemented by TurkStat between the years 2007 and 2014. The surveys focused on estimating household health care expenditure and the socioeconomic structure of households and individuals. Data on household consumption expenditures are compiled through interviews with household members and record books containing the daily expenditure of households held for a month. In the survey information on the socioeconomic status of the household in the first visit before the survey, a month is received and an expenditure book is left. During the visits within the survey month, data on consumption expenses of the household such as food, clothing, health, transport, communication, education, culture, recreation, housing, household goods, etc in the survey month are compiled. In the final visit at the end of the survey month, data on household members' employment and their income within the survey month is compiled. Each sample household in the survey is visited 8 times on average for one month. Total 77,313 observation data were associated/linked in these thirteen surveys and our analysis is based on the findings from these households.

Health expenditures data is collected against eight categories, i.e., (1) pharmacy products, (2) medical services (doctor of medical), (3) hospital services, (4) laboratory services, (5) other medical products, (6) medical auxiliary services, (7) dentistry, and (8) other services. All health expenditure items contained in the data set have been included in our study.

Besides, secondary data obtained from international databases were used in this study. Share of health expenditures in the gross domestic product (GDP), per capita health expenditures made by the public and gross domestic product per capita related data to the period 2007–2014 was taken from the organization for Economic Co-operation and Development (OECD) database on 12.11.2020. The reason for the lack of data on poverty rates, government's share in total health expenditure, and households' share in total health expenditure in Turkey over the years in the OECD database was taken from TurkStat. By Turkstat, the poverty rate in Turkey was calculated according to the relative poverty line based on income calculated using purchasing power parity (PPP).

### Analysis

Households' catastrophic health expenditures were estimated according to the methodology proposed by Xu and WHO (22). Percentages of health expenditure among total expenditure of households with positive health expenditure and households with catastrophic health expenditure between 2007 and 2014 have been evaluated using descriptive analysis. In the analysis, the expenditure ratio of households with positive health expenditure and households with catastrophic health expenditure according to HBS data were decomposed and evaluated separately. Data on health expenditures per capita, GDP per capita, and poverty rate obtained from OECD and TurkStat databases were evaluated by applying descriptive analysis. Findings have been interpreted in light of the health policies in practice between 2007 and 2014.

## Results

### Health Expenditure Ratios of Households With Positive Health Expenditure

**Figure 1:**
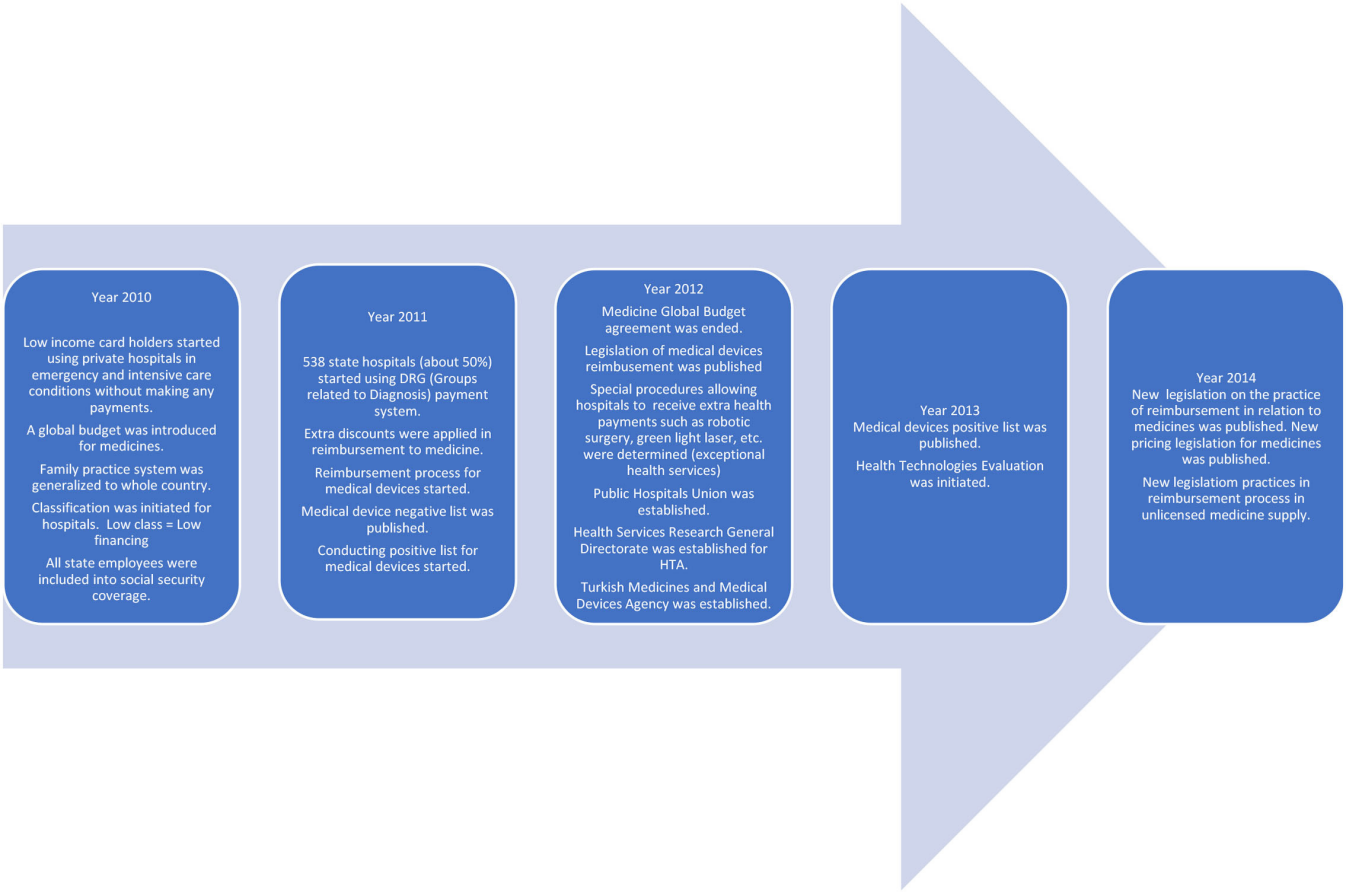
Health Policies that may have an impact on Health Access and Expenditures between 2010 and 2014 (25, 26). Figure 1 is prepared depending on the literature.

As a result of the Health Transformation Project in practice in the Turkish healthcare system since 2003, major changes have been done in all layers of the health system. Some of the policies and years of implementation (25) that may impact health access and health expenditures between 2010 and 2014 have been listed in Figure 1.

Seventy seven thousand three hundred thirteen observations were made with data from household visits during the month the study was conducted. The distribution of these observations by years is listed in Table 1.

**Table 1:** TURKSTAT HBS distribution of observations by years.

**Year**	**Number of observations**	**Percentage in total observations**
2007	8,548	11.06
2008	8,550	11.06
2009	10,046	12.99
2010	10,082	13.04
2011	9,918	12.83
2012	9,987	12.92
2013	10,060	13.01
2014	10,122	13.09
**Total**	**77,313**	**100**

According to the evaluation of households with positive health expenditure, average expenditure values of those with positive expenditure spending for pharmacy products, medical services (doctor of medical), hospital services, dentistry, and laboratory services have decreased. However, an increase has been observed in average expenditure values of those with positive expenditure spending on medical products, medical auxiliary products, and other services (Table 2).

**Table 2:** Health expenditure ratios of households with positive health expenditure (%).

	**2007**	**2008**	**2009**	**2010**	**2011**	**2012**	**2013**	**2014**
Pharmacy products	13.56 (29.00)	11.15 (21.68)	11.13 (24.66)	10.24 (24.00)	10.09 (18.99)	9.243 (20.48)	9.266 (20.25)	8.641 (13.85)
Other medical products	5.782 (15.27)	6.482 (16.07)	7.237 (28.43)	7.146 (16.13)	7.780 (15.71)	8.919 (18.59)	10.76 (37.98)	10.20 (28.35)
Devices and equipment used in treatment	62.34 (110.6)	49.30 (93.45)	46.39 (72.07)	51.98 (121.0)	50.58 (90.00)	45.88 (78.35)	45.33 (62.31)	60.30 (166.9)
Medical services (doctor of medical)	47.13 (51.42)	42.47 (53.46)	26.34 (97.71)	17.81 (41.05)	16.27 (28.94)	14.45 (26.62)	11.67 (25.35)	13.24 (25.21)
Dentistry services	146.6 (301.0)	105.6 (154.3)	104.6 (157.0)	42.73 (66.00)	49.95 (90.95)	49.53 (146.9)	40.42 (93.71)	42.63 (89.72)
Laboratories and x-ray centers	52.45 (93.27)	50.40 (85.51)	37.46 (60.82)	44.84 (127.4)	38.94 (130.6)	36.40 (82.13)	36.77 (60.11)	37.10 (69.46)
Medical auxiliary services	25.73 (66.80)	24.90 (56.74)	34.13 (86.48)	45.71 (97.72)	59.40 (143.3)	42.11 (88.36)	40.94 (100.6)	46.15 (117.9)
Other services	42.40 (76.81)	40.58 (78.00)	45.09 (74.73)	27.99 (66.35)	22.25 (42.38)	43.01 (84.77)	36.69 (55.89)	42.65 (68.18)
Hospital services	304.7 (612.1)	226.1 (717.5)	148.2 (247.1)	44.62 (103.5)	39.20 (73.10)	49.02 (121.6)	36.31 (72.98)	45.91 (109.2)
Total health expenditure (real)	39.43 (127.3)	33.11 (128.7)	31.33 (98.74)	33.37 (81.29)	33.20 (74.85)	32.53 (87.66)	32.56 (68.96)	35.66 (86.52)

### Health Expenditure Ratios of Households With Catastrophic Health Expenditure

Evaluation of change in the ratio of health expenditures according to categories of households with catastrophic health expenditure to the total spending by years, revealed a decrease in the ratio of health expenditures to total expenditures of individuals with positive spending for pharmacy products, medical services (doctor of medical), dentistry services, laboratory, and x-ray services. However, the ratio of health expenditures to total expenditures has increased in individuals with positive spending for other medical products, devices, and equipment used in treatment, auxiliary medical services, and hospital services (Table 3).

**Table 3:** Distribution by years, of the ratio of health expenditures by categories to total expenditures in households with catastrophic health expenditure.

	**2007**	**2008**	**2009**	**2010**	**2011**	**2012**	**2013**	**2014**	**Total**
Ratio of expenditures on pharmacy products to total health expenditures	21.29 (33.29)	17.95 (31.38)	17.23 (31.93)	15.32 (29.43)	8.127 (20.13)	8.130 (24.08)	21.57 (34.14)	7.732 (22.83)	15.71 (29.88)
Ratio of expenditures on devices and equipment used in treatment to total health expenditures	5.369 (19.74)	4.350 (19.96)	3.452 (17.66)	10.59 (29.88)	14.33 (34.56)	7.439 (25.83)	8.468 (25.36)	9.616 (26.56)	7.227 (24.19)
Ratio of expenditures on medical services (doctor) to total health expenditures %	21.64 (30.29)	20.05 (32.09)	19.96 (34.00)	23.09 (33.80)	12.23 (22.82)	2.610 (5.946)	12.86 (24.69)	9.130 (16.64)	16.96 (28.81)
Ratio of expenditures on dentistry services to total health expenditures %	18.84 (38.00)	13.73 (32.97)	20.32 (39.77)	6.904 (25.11)	23.52 (40.32)	12.79 (31.80)	8.465 (25.01)	17.62 (38.02)	15.83 (35.14)
Ratio of expenditures on laboratories and x-ray centers to total health expenditures	6.198 (16.36)	7.408 (18.83)	4.153 (11.79)	6.628 (19.39)	5.566 (13.80)	7.239 (22.18)	6.377 (13.51)	5.862 (12.49)	6.035 (15.97)
Ratio of expenditures on auxiliary medical services to total health expenditures	3.323 (16.04)	2.041 (11.13)	6.107 (23.53)	4.676 (19.24)	7.126 (26.17)	21.10 (39.11)	10.65 (28.76)	9.761 (29.67)	6.829 (23.83)
Ratio of expenditures on hospital services to total health expenditures %	18.53 (36.57)	28.48 (42.71)	25.25 (40.12)	24.68 (38.94)	29.07 (41.60)	27.69 (43.40)	26.11 (38.14)	35.83 (43.88)	26.03 (40.18)

**Figure 2:**
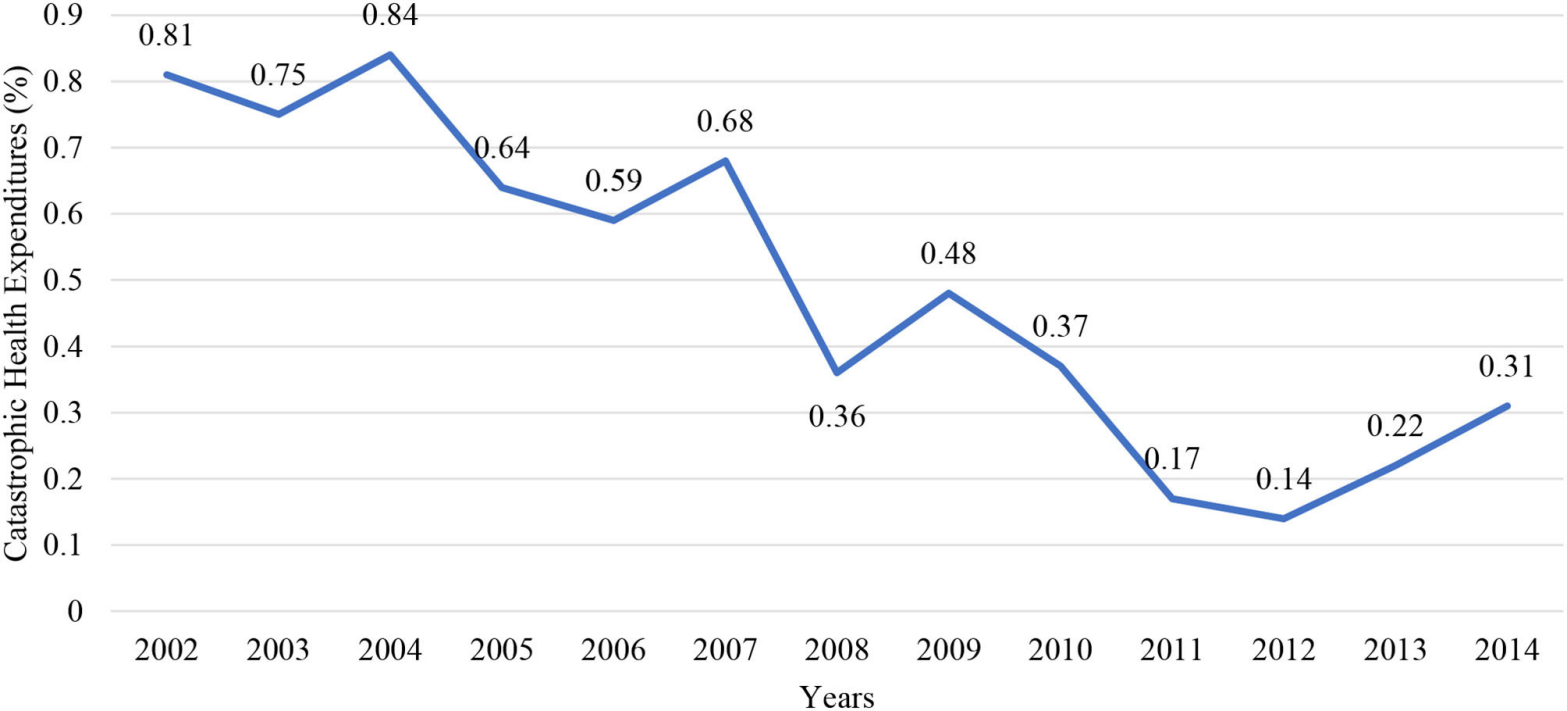
Catastrophic health expenditures by years (2002–2014) (24).

According to research conducted by TurkStat, the proportion of families spending catastrophically after the Health Transformation Project continued to fall until 2011. In 2002, 0.81% of the total household had catastrophic health expenses, while in 2011 this ratio decreased to 0.17%. But after these declines, the proportion of households spending on catastrophic health care in 2012 began to rise again. In 2014, the proportion of households spending on catastrophic health care reached 0.31% (Figure 2) (24).

**Figure 3:**
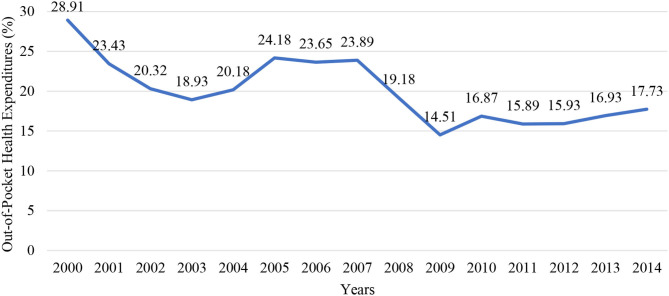
Percentage (%) of out of pocket health expenditures by years (2000–2014).

When out of pocket health spending in total health spending in Turkey is examined although a short-term increase was observed after the Health Transformation Program, there was a decrease between 2007 and 2011. The share of out of pocket health expenditures in total health expenditures continued to increase with fluctuations between 2012 and 2014 (Figure 3) (28).

When the results of the analysis are examined, it is seen that the health expenditure per capita covered by the public, and the GDP per capita increased in nominal value. Besides, the government's share of total health expenditure has also increased. Although health expenditure per capita has increased over the years, its share in GDP has decreased over the years. Besides, the poverty rate has been decreasing over the years (Table 4) (27).

**Table 4:** Health expenditures, GDP per Capita, poverty rate, and government's share in total health expenditure by years in Turkey (2007–2014) (27).

	**2007**	**2008**	**2009**	**2010**	**2011**	**2012**	**2013**	**2014**
GDP per capita	14,848	16,038	15,457	17,358	19,591	20,627	22,373	24,105
Health expenditures per capita covered by government	504.2	586.7	657.7	657.7	702.9	708.4	742.6	781.3
Share of health expenditures in GDP covered by government (%)	5.28	5.26	5.53	5.05	4.69	4.48	4.40	4.35
Poverty rates by poverty thresholds adjusted by PPP (%)	8.6	9.0	9.7	9.5	9.2	9.2	8.2	7.8
Government's share in total health expenditure (%)	67.8	73.0	81.0	78.6	79.6	79.2	78.5	77.4
Share of households in total health expenditures (%)	21.8	17.3	14	16.3	15.4	15.8	16.7	17.7

## Discussion

Growth of health expenditure is driven by several underlying issues: population birth rates, per-capita income, inflation, and so-called “excess growth” that is mostly explained by medical technology advances or increased patient demand for services. This “excess growth” is responsible for raising the share of health care in the national GDP and thus challenging fiscal sustainability (29). A percentage of general government expenditure on health and private expenditure on health per capita of total expenditure on health is very significant indicators of health expenditure (30). Especially public health expenditure to GDP ratio has increased middle income and in developing countries. But this ratio in Turkey is slightly declining over time. Although health care expenditure increase overall there is substantial concern about financial equity in health care financing.

The socioeconomic distribution of health expenditures varies between countries. However, it has been mentioned by Makinen et al. (31) that results between countries do not converge. Based on the findings of the study, Burkina Faso, Paraguay and Thailand have a regressive trend (rich households spend a lesser percentage of their total consumptions for health expenses compared to poor households) and Guatemala and South Africa has progressive trends. One of the most significant results of the study is that rich households are more likely to benefit from health services when they need compared to poor households. However, this may be due to poor households do not access any health services thinking that they cannot afford health expenses. Within this framework, the most significant drawback of the studies examining out of pocket health expenses is the inability to reflect the conditions where households refrain from using health services considering that they cannot meet the out of pocket health expenses required for health services usage of households. In other words, the level of forfeiting health services due to income restrictions shall be known.

According to the study by Xu et al. (16) studying on catastrophic health expense levels and their determinants for 59 countries, catastrophic health expenses are under 0.1% in countries like Canada, Czech Republic, Denmark, England, Germany, and France and catastrophic health expenses are high in some transition countries, middle income and low-income countries as well as some Latin American countries. For example, it is over 10% in Vietnam and Brazil and above 5% in Azerbaijan and Colombia. One of the significant aspects of the study is the authors researched the determinants and found a strong and positive relationship between catastrophic household percentage and the ratio of out of pocket health expenses to national health expenditures and reached the positive relation between catastrophic household percentage to the total share of health expenditure in GDP and percentage of household below the poverty line. The study clarifies that the highest catastrophic health expenditures which low-income groups generally are more subject to, should not belong to the lowest income group. This may also stem from selection bias.

In a 2012 study of 39,008 households in Iran, 3% of the total participants qualified as unemployed. The average monthly food expenditure for each household was US $ 174.7, the average consumption expenditure was US $ 606.4, and the average health expenditure was US $ 38.3. The share of health expenditures in total consumption expenditures is 6.3% and the share of these expenditures is 8.1% of the solvency for each household. The percentage of households exposed to catastrophic health expenditures was estimated at 2.8% with 2.1% for urban areas (Confidence Interval: 1.9–2.4%) and 3.4% for rural areas (Confidence Interval: 3.2–3.7%). Chi-Square and logistic regression analyses were performed to determine the factors affecting the household's exposure to catastrophic health expenditures. Multifactorial logistic regression analysis showed that drug addiction cessation and inpatient care spending had the greatest impact on households ' exposure to catastrophic health spending. Households using substance abuse cessation services and those using inpatient services were found to suffer between 13.33 and 11.84 times more catastrophic health expenses than households not using these services. It has emerged that drug spending has no impact on catastrophic health spending (32).

In 2018, a survey of 30,966 households in Peru used the National Household Survey to assess out-of-pocket catastrophic health expenditures. A logistic regression model was used for analysis. According to data from the regression model, health care services that have an impact on catastrophic health spending are divided into three groups. The catastrophic rate of health spending from the first group to the third group shows a decline. Medical tests, surgery, and drugs are in the first group. In the second group; hospital admission, outpatient treatment, dental and eye health are included. The third group includes child care, maternity care, contraceptives, and rehabilitation services (33).

In a study conducted by Narci et al. in Turkey in 2015, it was observed that the average household's monthly total consumption expenditure and payment capacity increased by about 30 and 42%, respectively, from 2004 to 2010. However, catastrophic spending households have a rate below 1%, even if it has increased over the years. The study found that the factor that led to an almost 9-fold increase in 2010 compared to 2004 was households paying for inpatient health services. In general, household income, number of people in the household, level of education, presence of members under 5 years of age, health insurance, payments for disabled members, and inpatient care members were found to be statistically significant determinants of catastrophic health expenditures (34).

In light of the preliminary analyses, our analysis shows that expenditures of households with health expenditure have decreased both in value and ratio in pharmacy, doctor, dentistry, and devices and equipment used in treatment, respectively from 13.56 to 8.641; from 47.13 to 13.24, from 146.6 to 42.63, and from 62.34 to 60.30 as a result of changes in health policies in Turkey. However, expenditures on other medical products, auxiliary medical services, and other services have increased both in ratio and in values respectively from 5.782 to 10.20; from 25.73 to 46.15, and from 42.40 to 42.62. Spending on hospital services has increased in ratio despite a decrease in values. In other words changes in the expenditure on medical products, devices and equipment used in treatment, other medical products, auxiliary medical services, other services, and hospital services may have contributed to the increase in the number of households with catastrophic health expenditures.

According to the statements of the Ministry of Health, the second phase of the Health Transformation Program has been initiated. In the second phase focus will be on sustainability and quality of the health services. Access to health is no longer an issue with the first phase of the project and policies on improving the quality have been implemented with the second phase. This can be assumed to increase health expenditures, naturally in Turkey. The main focus of the second phase is to ensure the quality and sustainability of health services. Ministry of Health has released to the press that it will lead new policies for this purpose.

An overview of the impact of the health policies reveals that medicine expenditures have decreased both for household and public health expenditures. With several implementations on pharmaceutical pricing and reimbursement policies to control expenditure that is criticized by the pharmaceuticals industry, the positive impact can be seen by the decrease in the spending on medicine for households spending on health. However, a great increase has been seen in spending on other medical products. Spending by the household on doctors has been seen to decrease and this can be explained by the full-day law policy that was implemented in 2010. Similarly, a dentist for all hospitals and counties project of the Ministry of Health can be the cause of the decrease in spending on dentistry services. Apart from these, it has been observed that ratios of spending on laboratories and x-ray centers and hospital services have decreased significantly and the main increase is in spending for devices and equipment used in the treatment and auxiliary medical services.

Hospital service with positive health expenditure is seen to decrease among health expenditure. The reasons for the increase in households with catastrophic health expenditure need further research. However, this can stem from the transfer of doctors that can perform specific surgeries to private hospitals as a result of full-day law and an increase in expenditures on specific diseases. Another reason can be the increase of additional fees of hospitals contracted with Social Security Institution to 200% of the price paid by the Institution. The changes in the reimbursement system for medical devices in recent years can be the cause of the increase in medical device expenditures. It can also stem from the fact that the proportion repaid by the Social Security Institution is not sufficient to purchase said products and patients need to purchase these products themselves for the treatments in parts of private hospitals and university hospitals.

As a result of the analysis, although health expenditures per capita covered by the public in Turkey have increased in nominal value over the years, the share of health expenditures in GDP has decreased as a ratio. The proportion of public spending on health care decreased from 5.28 to 4.35% between 2007 and 2014. Besides, the poverty rate fell from 8.6 to 7.8%, an improvement of close to 1%.

The government's share in total health expenditures has increased regularly between 2007 and 2011 but has started to decline from 2012. In connection with these rates, the share of households in total health expenditures decreased between 2007 and 2011 and started to increase since 2012. A decrease in the government's share in health expenditure may cause households and individuals to make catastrophic health spending. However, there is no increase in the overall poverty rate.

## Conclusion

As a result of the Health Transformation Program in practice in the Turkish healthcare system since 2003, major changes have been done in all layers of the health system. The impact of these changes on health expenditures has been evaluated in numerous studies. Many policies such as including green card holders, family practice systems, management of state hospitals, medical device reimbursement systems, and health technologies assessment methods into the process and arrangement of medicine pricing and reimbursement processes have been implemented. Some of the policies and years of implementation that may impact health access and health expenditures between 2010 and 2014. The study strives to discuss the possible policy reasons for the observed effects.

The study has a limitation as the data examined in the study covers only the years 2007–2014 only with descriptive analysis. The situation should be re-evaluated in further studies with current and longer interval data with statistical analysis to understand the difference and reason for the difference. Another limitation of the analysis is the lack of expenditures of the other indirect health-related expenditures like transportation, etc. However, this is the only available data set for Turkey that can be used for this kind of analysis. The analysis should be re-evaluated by including other indirect health-related expenditures.

The analysis shows a preliminary finding for further analysis. Although there is no increase in the overall poverty rate when the rates are examined, it can be said that individuals are suffering from the financial burden of catastrophic health expenditures due to the decrease of government healthcare spending and increase of household healthcare spending per capita in the given timeline. Further analysis should be conducted with the new and updated data availability, to compare the findings and validate the change of catastrophic health expenditure, government & household healthcare spending, and overall poverty rate.

The increase in the public share of total health expenditures between 2007 and 2011 naturally led to a decrease in households' out-of-pocket spending. Since 2012, the share of the public sector has started to decrease and the share of the household has started to increase. It can be said that this may lead to catastrophic health expenses. However, it is not clear why especially catastrophic health expenses increased in the expenditures of medical products, auxiliary medical services, and other services but decreased in expenditures of pharmacy, doctor, dentistry, and devices and equipment used. The reasons for the increase or decrease in households with catastrophic health expenditure and the changes observed in ratios of health expenditures shall be evaluated with further studies for getting the right policy decisions to be implemented.

## Data Availability Statement

Publicly available datasets were analyzed in this study. This data can be found here: http://www.tuik.gov.tr.

## Author Contributions

GK: data collection, literature review and writing, and data analysis. GO and ZÇ: idea, interpretation, revision, and writing. All authors contributed to the article and approved the submitted version.

## Conflict of Interest

GK is the founder and chief executive officer of the company ECONiX Research Analysis and Consultancy Plc. The remaining authors declare that the research was conducted in the absence of any commercial or financial relationships that could be construed as a potential conflict of interest.

## Abbreviations

FP: Financial Protection
GDP: Gross domestic product
OECD: Organization for Economic Co-operation and Development
PPP: Purchasing Power Parity
SDG: Sustainable Development Goal
TURKSTAT: Turkish Statistics Institute
UHC: Universal Health Coverage
WHO: World Health Organization.
